# A hospital-based therapeutic food pantry study for people living with cancer in New Orleans

**DOI:** 10.1007/s00520-023-08171-x

**Published:** 2023-11-20

**Authors:** Ting Luo, Bilikisu Elewonibi, Donna Williams

**Affiliations:** 1 Moores Cancer Center, University of California San Diego, La Jolla, CA 92122 USA; 2 Epidemiology and Population Health, School of Public Health, Louisiana State University Health Sciences Center-New Orleans, New Orleans, LA 70112 USA; 3 Behavioral and Community Health Sciences, School of Public Health, Louisiana State University Health Sciences Center-New Orleans, New Orleans, LA 70112 USA

**Keywords:** People living with cancer, Food pantry, Food insecurity, Gender differences, Fruit and vegetable

## Abstract

**Introduction:**

Food pantries have the potential to improve health outcomes and quality of life for individuals living with cancer. Gender has been linked to certain cancer symptoms and dietary patterns. Nevertheless, the extent of research on the utilization of food pantries among this population, particularly with regard to gender differences, remains limited. The objective of this study is to explore the demographic characteristics and gender differences in quality of life, as well as the impact of cancer on the lives of individuals who utilize food pantry services.

**Methods:**

Between February 26, 2019 and July 24, 2022, 400 people living with cancer were eligible to participate the University Medical Center New Orleans (UMC) food pantry. Participants were asked to provide demographic information and completed two health assessments related to the challenges in daily activities, nutrition, and mental health.

**Results:**

The study participants had a mean age of 54.1, and the majority of the participants were female. More than half of the participants did not have access to a vehicle or use public transportation to access grocery stores. People living with cancer reported several quality of life issues, with the most prevalent challenges being interference of cancer with work, lack of energy, difficulty affording food, pain, and sleep problems. Additionally, less than half of the patients reported consuming fruits and vegetables on a daily basis, and males were found to be less likely to consume them compared to females.

**Discussion:**

The current study sheds light on the characteristics and quality of life of individuals who utilize UMC food pantry services, as well as the impact of cancer on their lives. The findings reveal a gender disparity in fruit and vegetable consumption, with male individuals living with cancer reporting lower levels of consumption.

**Implications for research and practice:**

Identifying and addressing food insecurity among people living with cancer are necessary. Meanwhile, partnerships with community organizations may be valuable in finding ways to assist cancer survivors in returning to work. Future studies could also focus on encouraging fruit and vegetable consumption, particularly among male individuals living with cancer.

## Introduction

Malnutrition is a prevalent issue among individuals living with cancer, and is often linked with poor treatment outcomes, lower quality of life, morbidity, and mortality [1–3]. The rate of malnutrition among people living with cancer ranges from 40 to 80% [4, 5], and about 20% of people living with cancer die as a result of malnutrition rather than cancer complications [6]. Cancer tumors, like pain, stress, or depression, can interfere with a person’s appetite [6, 7], and cancer treatments such as chemotherapeutic, radiotherapeutic, and surgical regimens may also lead to changes in eating habits and the desire to eat [5]. Cancer treatments tax the body and sometimes lead to nausea, vomiting, and loss of appetite making adequate nutrition even more important during treatment [8, 9]. Furthermore, malnutrition has been shown to be negatively associated with treatment tolerance by increasing side effects and adverse reactions thus reducing 5-year survival rates [5]. Finally, malnutrition can result in an extended hospital stay, leading to increased healthcare costs among individuals living with cancer [10, 11].

Food insecurity is a major contributor to malnutrition in the United States (U.S.), defined as a lack of consistent access to enough food to lead an active and healthy life [12]. According to the U.S. Department of Agriculture (USDA) 2020 report, about 10% of Americans and about 15% of Louisiana residents, experienced food insecurity [12]. This issue is more prevalent in low-income households, with an estimated 35% of Americans with household incomes below the federal poverty line experiencing food insecurity [12]. People living with cancer are particularly vulnerable to the effects of food insecurity due to the nature of the disease and the intensive treatment protocols [13]. A systematic review estimated the prevalence of food insecurity among people living with cancer at 17.4%, with an increasing trend [13]. Other national-level studies estimate the prevalence of food insecurity among people with cancer histories is between 8.4 and 22.7% [14, 15]. In addition, cancer diagnoses and treatment options are associated with social, occupational, and financial stressors, and any subsequent loss of income may further exacerbate food insecurity [16].

Louisiana is considered one of the hungriest and poorest states. According to American Health Ranking 2021, Louisiana ranked 48th in food insecurity, 44th in nutrition, 47th in obesity, and 50th in economic resources [17]. Feeding America states 718,360 people are facing hunger in the state, an estimated 1 in 6 Louisianan people [18]. The food insecurity in New Orleans is even worse than the Louisiana average as about 1 in 5 New Orleanians are food insecure, and getting food on the table regularly is a constant challenge for a good amount of the population [19, 20]. Food insecurity was identified as a patient major issue in a 2015 Community Health Needs Assessment (CHNA) conducted at the University Medical Center New Orleans (UMC), involving 709 patients of all types. The survey also reported that 73% of these patients lived below 150% of the poverty level, 49% of patients found it difficult to afford food or nutrition supplements, and only 13% consumed fruit and vegetables every day [21].

Nutrition screening and interventions have the potential to improve health outcomes and quality of life for individuals living with cancer [22]. Prior research indicates that incorporating nutritional intervention into comprehensive cancer management is beneficial [22]. For instance, studies have demonstrated that nutritional counseling and dietary supplements, such as omega-3 fatty acids (eicosapentaenoic acid (EPA) and/or docosahexaenoic acid (DHA)), can mitigate drug-induced side effects and enhance therapeutic efficacy for those affected by breast cancer [22]. Nutritional intervention may help cancer patients reduce fatigue and increase their quality of life by addressing clinical and psychological factors, such as anxiety, depression, self-care capacity, and immune responses [6, 23]. Moreover, a randomized controlled trial shows that a 12-week intervention with soy supplementation can improve measures of quality of life for individuals living with prostate cancer [24].

In light of the observations at UMC and the evidence supporting nutritional interventions, the therapeutic food pantry was established at UMC in 2018 as Louisiana’s first hospital-based food pantry. This initiative resulted from a multi-sector partnership involving UMC, Second Harvest Food Bank, Louisiana Cancer Prevention & Control Programs (LCP), Baptist Community Ministries, Methodist Health System Foundation, and the Blue Cross and Blue Shield of Louisiana Foundation. The pantry offers high-quality, fresh, and nutritious food to individuals living with cancer who may be experiencing food insecurity and malnutrition. Additionally, the pantry aids those unable to secure groceries independently.

The objective of this study is to describe the characteristics of individuals living with cancer who utilize the food pantry services at UMC. We examine demographic characteristics, quality of life, nutrition challenges, activities, and mental health of food pantry participants. As gender has been linked to certain cancer type (like prostate cancer in men and breast cancer in women), certain cancer symptoms (males had higher rates of dysphagia, hoarseness, and weight loss, whereas females reported higher rates of early satiety, nausea, vomiting, and anxiety) [25], dietary patterns [26–28], and dietary preferences [29], we also evaluate gender differences in the assessed indicators of this study.

## Methods

### Study data and sample

The therapeutic food pantry is situated in the Cancer Center office at UMC, equipped with shelving and refrigeration units. Food is supplied by the Second Harvest Food Bank of Greater New Orleans and Acadiana. Two dietitians at the Cancer Center collaborate closely with Second Harvest to maintain a well-stocked pantry. Patients can register for the food pantry during a visit, and they are accompanied by one of the dietitians to select food items. Data for this study was obtained from patient registration information at the food pantry. Individuals living with cancer and having family incomes below 130% of the federal poverty level qualify for food pantry registration, mirroring Medicaid eligibility. This study utilizes data from February 26, 2019 to July 24, 2022, with 406 cancer patients registered for pantry services. All but six registered patients were included in the analysis; one patient had incomplete demographic information, while the other five submitted blank surveys. All materials and procedures were approved by the Internal Review Board of Louisiana State University Health Sciences Center (#10337).

### Tools and measures

Demographic and social determinants of health indicators, such as age, sex, address, transportation to a store, and time to store, were gathered during the registration period. Transportation to a store was assessed using an open-ended question: “How do you usually go to a store?” Patients who responded with anything other than “Drive a car,” “Get a ride,” “By bike,” “Take public transportation,” “Walk,” or “I do not shop at a health food store” were categorized as “Other.” Time to a store was measured with the question: “How long does it usually take you to get to a store?” Response options included “0–14 min,” “15–29 min,” “30–35 min,” and “1 h or longer.”

Two health assessment tools were administered by nutritionists during patient intake and provided to all patients who consulted with the nutritionists. The Cancer Rehabilitation Evaluation System–short form (CARES‐SF) was adapted and shortened from 60 to 17 questions [30–32]. These questions assess quality of life across three categories: daily activities, nutrition, and mental health. The daily activities section included seven indicators: difficulty in bending or lifting, difficulty in doing chores, difficulty in bathing and grooming, difficulty in planning activities, lack of energy, cancer interfering with the ability to work, and pain. The nutrition section featured five indicators: difficulty in affording food, food unappealing, difficulty in gain weight, clothes not fit, and discomfort with body change. Lastly, the mental health section comprised five indicators: anxiety, sleep problems, difficulty in concentrating, difficulty in asking others for help, and difficulty in telling others about cancer. For each indicator, patients were asked “How much does it apply to you?”. Response options were “not at all,” “a little,” “fair amount,” “much,” and “very much.” For analysis purposes, “not at all” and “a little” were combined as “No,” “fair amount,” “much,” and “very much” were grouped as “Yes.”

The second health assessment tool was adapted from the Short Form-8 Health Survey (SF-8) and included two additional questions beyond the original eight [33, 34]. The first five questions inquired if patients “had to limit physical activity,” “had difficulty in doing daily work,” “had to limit social activities,” “were kept from usual work,” and “were bothered by emotional problems” in the past 4 weeks. Response options were “Not at all,” “Very little,” “Somewhat,” “Quite a lot,” and “Couldn’t do.” “Not at all” and “Very little” were grouped as “No” while “Somewhat,” “Quite a lot,” and “Couldn’t do” were combined as “Yes.” The next two questions asked “how would you rate your health overall in the past 4 weeks” and “how much bodily pain have you had in the past 4 weeks.” Responses were recoded into two groups for analysis purposes. The last three questions were multiple choice questions that asked how cancer had impacted their nutrition, energy, and exercise during the past 4 weeks. Details on these questions, responses, and regrouping strategies can be found in the footnote of Table 1.

### Analysis

The patients’ demographic data and the two health assessment data were linked using unique patients IDs. Descriptive statistics were employed to examine the frequency of each health indicator. Chi-square tests were utilized to compare the differences in participants’ characteristics within a group and to investigate differences in each health indicator between males and females. The alpha level at 0.05 was considered statistically significant for all analyses. SAS (statistical analysis system) software 9.4 was used to run the analysis.

## Results

### Study participants

Table 1 presents the characteristics of the 400 study participants. The mean age of participants was 54.1 years. Approximately 38% (*n* = 137) were 60–69 years old, and 32% (*n* = 117) were 50–59 years old. The study sample consisted of more females (56.5%, *n* = 227) than males. Over half of the participants (59.3%, *n* = 240) were from New Orleans. Participants’ modes of transportation to grocery stores varied: less than half reported driving a car (45.3%, *n* = 68), 29.3% reported getting a ride (*n* = 44), 7.3% reported walking (*n* = 11), 7.3% reported biking or taking public transportation (*n* = 11), and the rest reported “other” (4.7%, *n* = 7). Six percent of participants reported not shopping at a grocery store (*n* = 9). About 40% of participants reported it took them 0–14 min to get to a store, another 40% took 15–29 min, approximately 10% reported taking 30–59 min, and the remaining participants took 1 h or longer. A statistically significant difference was found in the age group distribution. The differences were primarily observed in the 60–69 age group, which had more males (51.6%) than females (36.7%).

**Table 1:** Characteristics of University Medical Center food pantry study participants (*N* = 405)

Variables	Number (%)	*p* value^1^	Female (%)	Male (%)	*p* value^2^
Age (mean, SD)	54.1 (53.6)				
Age		< 0.0001			0.05
= < 40	30 (8.2)		16 (7.7)	14 (8.9)	
40–49	51 (13.9)		36 (17.2)	14 (8.9)	
50–59	117 (31.8)		71 (34.0)	45 (28.7)	
60–69	137 (37.2)		67 (32.1)	70 (44.6)	
> = 70	33 (9.0)		19 (9.1)	14 (8.9)	
Sex		0.0002			
Female	228 (56.6)				
Male	175 (43.5)				
City		0.0083			0.74
New Orleans	240 (59.3)		133 (58.3)	105 (60.0)	
Others	165 (40.7)		95 (41.7)	70 (40.0)	
Transportation to a store		< 0.0001			0.31
Car	68 (45.3)		42 (47.7)	25 (41.0)	
Get a ride	44 (29.3)		29 (33.0)	15 (24.6)	
Bike or public transportations	11 (7.3)		4 (4.6)	7 (11.5)	
Walk	11 (7.3)		4 (4.6)	7 (11.5)	
I do not shop at a healthy food store	9 (6.0)		5 (5.7)	4 (6.6)	
Other	7 (4.7)		4 (4.6)	3 (4.9)	
Time to store		< 0.0001			0.47
0–14 min	60 (42.6)		38 (45.8)	21 (36.8)	
15–29 min	62 (44.0)		35 (42.2)	27 (47.4)	
30–59 min	13 (9.2)		8 (9.6)	5 (8.8)	
1 h or longer	6 (4.3)		2 (2.4)	4 (7.0)	

^1^*p* value: tests the differences within groups

^2^*p* value: tests the differences between males and females

The variables with numbers that do not sum up to 405 indicate the presence of missing data for those particular variables

### Quality of life

Table 2 displays the results of the quality of life assessment. A significant number of participants reported challenges in daily activities, with 74.2% indicating that cancer interfered with their ability to work, 73.3% reporting having much less energy than they used to, and 69.3% experiencing pain. Approximately half of the participants reported difficulty doing chores (58.5%), having trouble bending or lifting (57.1%), or struggling with planning activities (49.2%). However, 75% of participants reported no difficulty in bathing and grooming. Over 71% of participants experienced difficulty affording food. Around 60% reported that clothes did not fit and felt uncomfortable with body changes, respectively. Less than half of the participants found food unappealing (42.0%) and had difficulty gaining weight (47.3%).

**Table 2:** Self-reported quality of life for University Medical Center food pantry survey respondents (*N* = 400)

	Overall		Female		Male		*p* value
	No^1^ (%)	Yes^2^ (%)	No (%)	Yes (%)	No (%)	Yes (%)	
Activities							
Difficulty in bending or lifting	**42.9**	**57.1**	41.3	58.7	45.0	55.0	0.47
Difficulty in doing chores	**41.5**	**58.5**	38.0	62.0	46.2	53.8	0.10
Difficulty in bathing and grooming	**76.0**	**24.0**	74.9	25.1	77.5	22.5	0.55
Difficulty in planning activities	**50.8**	**49.2**	54.8	45.2	45.5	54.5	0.07
Lack of energy	**26.7**	**73.3**	29.0	71.0	23.5	76.5	0.22
Cancer interfering with the ability to work	**25.9**	**74.2**	29.5	70.5	21.1	78.9	0.06
Pain	**30.7**	**69.3**	30.5	69.5	31.0	69.0	0.92
Nutrition							
Difficulty in affording food	**28.7**	**71.3**	31.5	68.5	25.0	75.0	0.16
Food unappealing	**58.0**	**42.0**	56.8	43.2	59.5	40.5	0.58
Difficulty gaining weight	**52.7**	**47.3**	58.6	41.4	45.0	55.0	0.01**
Clothes do not fit	**39.9**	**60.1**	41.4	58.6	37.9	62.1	0.49
Uncomfortable with body change	**41.2**	**58.8**	39.6	60.5	43.3	56.7	0.46
Mental health							
Anxious	**46.4**	**53.6**	47.3	52.7	45.2	54.8	0.68
Sleep problems	**35.1**	**65.0**	35.7	64.3	34.1	65.9	0.75
Difficulty in concentrating	**49.6**	**50.4**	49.1	50.9	50.3	49.7	0.81
Difficulty in asking others to do things for me	**44.2**	**55.8**	41.2	58.8	48.2	51.8	0.17
Difficulty in telling others about the cancer	**57.7**	**42.3**	60.2	39.8	54.3	45.7	0.25

Participants were asked, “How much does it apply to you?” Response options included “Not at all,” “A little,” “Fair amount,” “Much,” and “Very much

*Indicates significance < 0.05; **Indicates significance < 0.01; ***Indicates significance < 0.001

^1^ “No” includes not at all and a little

^2^ “Yes” includes a fair amount, much, and very much

Regarding mental health challenges, 65% of participants reported sleep problems. Slightly more than half experienced anxiety (53.6%), difficulty asking others for help (55.8%), and trouble concentrating (50.4%). Fewer than half of participants reported difficulty discussing cancer with others (42.3%). A statistically significant difference was found between males and females (*p* = 0.01) concerning weight gain. Males (55.0%) were more likely to report problems gaining weight than females (41.1%).

### Physical, mental, and overall health in the past 4 weeks

Table 3 presents participants’ physical, mental, and overall health for the 4 weeks prior to the survey. Approximately 75% of participants reported having to limit physical activities, 74% indicated difficulty in performing daily work, and 70% mentioned having to limit social activities. Around 62% of participants were kept from their usual work, and 57% were bothered by emotional problems. Additionally, 65% of participants experienced body pain, and 50% reported poor overall health. No statistically significant differences between males and females were found in these factors.

**Table 3:** Self-evaluation of physical, mental, and overall health during past 4 weeks (*N* = 400)

	Overall		Female		Male		*p* value
	No^1^ (%)	Yes^2^ (%)	No (%)	Yes (%)	No (%)	Yes (%)	
Had to limit physical activities^3^	**25.2**	**74.8**	24.0	76.0	26.8	73.2	0.52
Difficulty in doing daily work^4^	**26.4**	**73.6**	28.4	71.6	23.6	76.4	0.29
Had to limit social activities^5^	**30.5**	**69.5**	30.6	69.4	30.4	69.6	0.98
Kept from usual work^6^	**37.5**	**62.3**	38.3	61.8	37.0	63.0	0.81
Bothered by emotional problems^7^	**43.5**	**56.5**	41.7	58.3	46.0	54.0	0.41
Body pain^8^	**34.8**	**65.2**	31.8	68.2	38.8	61.2	0.16
Overall health^9^	**Good**^**10**^** (%)**	**Poor**^**11**^** (%)**	Good (%)	Poor (%)	Good (%)	Poor (%)	
	**50.0**	**50.0**	50.8	49.2	49.0	51.0	0.79

^1^ “No” includes not at all and very little. (for body pain: “No” includes none, very mild, and mild.)

^2^ “Yes” includes somewhat, much, and very much. (for body pain: “Yes” includes moderate, severe, and very severe.)

^3^ “Had to limit physical activities”: during the past 4 weeks, how much did physical health problems limit your usual physical activities?

^4^ “Difficulty in doing daily work”: during the past 4 weeks, how much difficulty did you have done your daily work, both at home, and away from home, because of your physical health?

^5^ “Had to limit social activities”: during the past 4 weeks, how much did your physical health or emotional problems limit your usual social activities with family or friends?

^6^ “Kept from usual work”: during the past 4 weeks, how much did personal or emotional problems keep you from doing your usual work, school, or other daily activities?

^7^ “Bothered by emotional problems”: during the past 4 weeks, how much have you been bothered by emotional problems (such as feeling anxious, depressed, or irritable?); the response to this question was “Extremely” instead of “Could not do”

^8^ “Body pain”: during the past 4 weeks, how much bodily pain have you had?

^9^ “Overall health”: during the past 4 weeks, overall, how would you rate your health?

^10^ “Good” includes excellent, very good, and good

^11^ “Poor” includes somewhat, poor, and very poor

### Nutrition, energy, and exercise in the past 4 weeks

Table 4 presents participants’ nutrition, energy, and exercise levels for the 4 weeks prior to the survey. Approximately half of the participants reported not consuming fruits and vegetables on a daily basis (55%), lacking energy (52%), and having difficulty meeting CDC recommendations for minimal exercise (49%). A statistically significant difference in fruit and vegetable intake was found between males and females (*p* = 0.01). Females (60.5%) were more likely to report consuming fruits and vegetables on a daily basis compared to males (47.9%).

**Table 4:** Self-evaluation of nutrition, energy, and exercise during past 4 weeks (in percentage) (*N* = 400)

	Overall		Female		Male		*p* value
Fruit and vegetable Intake^7^	**At least once a day**^**8**^	**Less than once a day**^**9**^	At least once a day	Less than once a day	At least once a day	Less than once a day	
	**44.9**	**55.1**	39.5	60.5	52.1	47.9	0.01**
Having energy^10^	**Yes**^**11**^	**No**^**12**^	Yes	No	Yes	No	
	**47.8**	**52.2**	48.4	51.6	47.0	53.0	0.78
Met CDC recommended exercise^13^	**Yes**^**14**^	**No**^**15**^	Yes	No	Yes	No	
	**51.4**	**48.6**	55.5	44.5	46.1	53.9	0.07

*Indicates significance < 0.05; **Indicates significance <0.01; ***Indicates significance <0.001

^7^ “Fruit and vegetable intake”: during the past 4 weeks, how often do you eat fruits and vegetables?

^8^ “At least once a day” includes twice or more per day and once per day

^9^ “Less than once a day” includes 5–6 times per week, 2–4 times per week, and once per week

^10^ “Having energy”: during the past 4 weeks, how much energy do you have?

^11^ “Yes” includes very much and some

^12^ “No” includes a little and none

^13^ “Met CDC recommended exercise”: during the past 4 weeks, did you exercise 30 min a day, 5 days a week?

^14^ “Yes” includes always and sometimes

^15^ “No” refers to never

## Discussion

This study examines the demographic characteristics and quality of life indicators of individuals living with cancer who utilized the food pantry services at UMC. It also investigates the impact of cancer on their physical, mental, and overall health, nutrition, energy, and exercise in the past 4 weeks. Our study found that approximately 60% of the participants took more than 15 min to get to a grocery store, and 55% did not drive a vehicle. A previous study reported that long distances between the place of residence and food purchase locations were associated with a lack of nutrient-dense foods for elderly people, such as fish, fish products, beef, fruits, and vegetables [35]. Furthermore, the study pointed out that distance to food stores may influence people’s feelings of food insecurity [35]. Individuals living with cancer often have hospital appointments and lower energy levels, which can exacerbate distance-related problems when they need to get a ride from someone, use public transportation, or ride a bike. A lack of transportation often prevents individuals from accessing food pantries or nutrient-dense foods [36]. Although the food pantry in this study is located at the hospital where patients receive treatment, it may be challenging for patients without a vehicle to transport the food provided home. Thus, offering transportation services or delivery to individuals living with cancer could be a potential solution to address transportation issues.

This study also found that individuals living with cancer faced numerous quality of life challenges, with the five most prevalent ones being: interference with the ability to work (74.2%), lack of energy (73.3%), difficulty in affording food (71.3%), pain (69.3%), and sleep problems (65.0%). A previous study conducted in Western Europe, which focused on patients with digestive cancer, found that they encountered similar challenges at the start or 3 months after treatment. These challenges included a reduction in energy, interference of cancer with their ability to work, and difficulties with sleep [32]. While difficulty in affording food was not among the top 10 challenges reported in the Western European study [32], our study found that difficulty in affording food ranked among the top 5 challenges for our participants. This disparity highlights certain circumstances and factors that can vary across different populations and regions. Research has shown that low income is the primary cause of food insecurity [12], and cancer makes food insecurity worse for low-income patients [37]. A previous study showed that 55% of food insecure cancer patients did not take prescribed medication due to affordability issues, compared to 12.8% of food secure patients [37]. Food-insecure patients may have difficulty complying with prescribed therapies because they may need to spend their money on food instead and reported higher rates of depression and nutritional risk, and lower quality of life, compared to food-secure patients [37]. As a result, patients’ quality of life and treatment outcomes may be affected in large part by food insecurity. An intervention study found that food pantry, food voucher plus pantry, and home grocery delivery plus pantry access all significantly improved food insecurity issues and treatment completion rates among people living with cancer [38]. The voucher plus access to a pantry had the highest treatment completion rate (94.6%), followed by grocery delivery plus pantry access (82.5%), and pantry access (77.5%) [38]. Therefore, identifying food insecure patients and developing appropriate intervention strategies to help them will aid in treatment compliance, ultimately enhancing their quality of life.

The most prevalent challenge among study participants was cancer interfering with the ability to work, with about 74.2% reporting it. The inability to work as a result of cancer has a negative impact on their income, which further adds to food insecurity. Ekwueme et al.’s study found that about 25% of cancer survivors who were employed at any time since diagnosis reported feeling less productive at work [39]. Compared to males, females were more likely to make changes in work due to cancer [39]. The study tools used here did not collect information on employment status; hence, we could not examine any differences in variables of interest by employment status. Other studies have shown that more than one in four cancer patients experience a decreased ability to work or are even unable to return to work [39, 40]. Future research may partner with community organizations to find ways to assist cancer survivors in returning to work, which could reduce the family income burden and alleviate food insecurity issues. Although a majority of respondents reported experiencing pain, difficulty bending or lifting, and performing chores, it is noteworthy that 58.8% of women and 51.8% of men expressed difficulty in asking others to assist them, indicating a prevalent hesitancy among both genders. This finding suggests that while the patients in our sample required assistance, they were reluctant to seek help. While outside the scope of this project, future studies could investigate interventions aimed at providing tangible social support in ways that align with patients’ reluctance to ask for assistance.

Finally, our study found that less than half of the patients consumed fruits and vegetables, which are usually considered to be nutrient-rich foods. We also observed that more male than female individuals living with cancer reported having fruit and vegetable on daily basis, results that have been observed in previous studies [41–43]. Cancer survivors’ diet plays an important role in their overall health, quality of life, and survival [5, 44]. When patients cannot obtain adequate or nutritious food, they may become malnourished, which can affect their ability to tolerate and respond to oncology treatment, increasing their risk of recurrence and death from cancer [5, 44]. Food insecure patients may not be able to afford nutrient-rich food that could help them receive optimal nutrition during treatment [37]. Food pantries can provide individuals living with cancer with nutrient-rich foods; hence, promoting food pantries may benefit cancer survivors, especially those who are food insecure.

This study has several limitations. Our study modified two commonly used measures of quality of life to meet our measurement purposes, which might compromise their original validity and reliability. However, it is worth noting that our study measured 17 indicators of quality of life, providing important insights into the quality of life in this study population. The information collected in this study was self-reported; hence, the percentages of health-related indicators, time to store, and transportation to store might have been underestimated or overestimated. Our study also lacks follow-up data and assessment, preventing us from evaluating the effectiveness of the UMC food pantry program on patient quality of life or nutrition. Fourth, the sex determined in our study was based on the data collection at UMC, which only included two categories (male and female) and did not include other gender identities. Furthermore, we did not collect information on other factors such as race, which may have influenced the results. In addition, the two dietitians who were employed at the food pantry were also the individuals responsible for data collection. This overlap could potentially introduce a bias, as they may have a vested interest in the success of the food pantry program.

## Conclusion and implications for public health

This study provides valuable information about the characteristics, quality of life, nutrition, and mental health of people living with cancer at UMC who utilized the food pantry. As a result of this evidence, we can better understand people living with cancer who participate in food pantries. Future studies with pre-post intervention outcome measurements are needed to further assess the utilization and impact of the food pantry program among people living with cancer. The results also highlight the need to identify food insecure individuals, develop appropriate intervention strategies to help them obtain nutritious food, and form partnerships with community organizations to find ways to assist cancer survivors in returning to work. Our data suggests that male individuals living with cancer were less likely to consume fruits and vegetables. Hence, future studies might benefit from encouraging fruit and vegetable consumption, especially among male individuals living with cancer.

## Data Availability

The datasets generated during and/or analyzed during the current study are available from the corresponding author on reasonable request.

## References

[1] Brinksma A, Sanderman R, Roodbol PF (2015). Malnutrition is associated with worse health-related quality of life in children with cancer. Support Care Cancer.

[2] Polański J, Jankowska-Polańska B, Uchmanowicz I, Chabowski M, Janczak D, Mazur G, Rosińczuk J (2017). Malnutrition and quality of life in patients with non-small-cell lung cancer. Pulmonary Care and Clinical Medicine.

[3] Loeffen EA, Brinksma A, Miedema KG, De Bock G, Tissing WJ (2015). Clinical implications of malnutrition in childhood cancer patients—infections and mortality. Support Care Cancer.

[4] Isenring E, Bauer J, Capra S (2003). The scored patient-generated subjective global assessment (PG-SGA) and its association with quality of life in ambulatory patients receiving radiotherapy. Eur J Clin Nutr.

[5] Lis CG, Gupta D, Lammersfeld CA, Markman M, Vashi PG (2012). Role of nutritional status in predicting quality of life outcomes in cancer—a systematic review of the epidemiological literature. Nutr J.

[6] Capra S, Ferguson M, Ried K (2001). Cancer: impact of nutrition intervention outcome - Nutrition issues for patients. Nutrition.

[7] Smith HR (2015). Depression in cancer patients: pathogenesis, implications and treatment. Oncol Lett.

[8] Rowbottom L, McDonald R, Turner A, Chow E, DeAngelis C (2016). An overview of radiation-induced nausea and vomiting. J Med Imaging Radiat Sci.

[9] Galindo DEB, Vidal-Casariego A, Calleja-Fernández A (2017). Appetite disorders in cancer patients: impact on nutritional status and quality of life. Appetite.

[10] Planas M, Álvarez-Hernández J, León-Sanz M, Celaya-Pérez S, Araujo K, García de Lorenzo A (2016). Prevalence of hospital malnutrition in cancer patients: a sub-analysis of the PREDyCES® study. Support Care Cancer.

[11] Zhang X, Edwards BJ (2019). Malnutrition in older adults with cancer. Curr Oncol Rep.

[12] USDA. Food Security and Nutrition Assistance. USDA Econimic Research Service. https://www.ers.usda.gov/data-products/ag-and-food-statistics-charting-the-essentials/food-security-and-nutrition-assistance/#:~:text=In%202020%2C%2089.5%20percent%20of,from%2010.5%20percent%20in%202019. Accessed 20 Apr 2022

[13] Coughlin SS (2021) Food insecurity among cancer patients: a systematic review. Int J Food Nutr Sci. 10.15436/2377-0619.21.3821

[14] Trego M, Baba Z, DiSantis K, Longacre M (2019). Food insecurity among adult cancer survivors in the United States. J Cancer Surviv.

[15] Charkhchi P, Fazeli Dehkordy S, Carlos RC (2018). Housing and food insecurity, care access, and health status among the chronically ill: an analysis of the behavioral risk factor surveillance system. J Gen Intern Med.

[16] Naughton MJ, Weaver KE (2014). Physical and mental health among cancer survivors: considerations for long-term care and quality of life. N C Med J.

[17] Rankings AsH. American’s Health Rankings Annual Report. https://www.americashealthrankings.org/explore/annual/measure/food_insecurity_household/state/LA. Accessed 15 May 2022

[18] America F. What hunger looks like in Louisiana. https://www.feedingamerica.org/hunger-in-america/louisiana. Accessed 15 May 2022

[19] Baldridge E. Hungry for change: a look into The New Orleans food crisis. Updated Dec 16, 2019. https://www.vianolavie.org/2019/12/16/hungry-for-change-a-look-into-the-new-orleans-food-crisis/#:~:text=1%20in%20every%206%20residents,the%20National%20School%20Lunch%20Program. Accessed 15 May 2022

[20] Blair C Persistent food insecurity clashes with New Orleans’ rep for glorious gastronomy. The lens focused on New Orlenas and The Gulf Coast. https://thelensnola.org/2018/12/14/persistent-food-insecurity-clashes-with-new-orleans-pride-in-our-vibrant-food-culture/. Accessed 15 May 2022

[21] Umbach T. University Medical Center New Orleans Community Health needs assessment. https://www.lcmchealth.org/sub/59170/documents/UMC_2015_Community_Health_Needs_Assessment_-_Final.pdf. Accessed 18 May 2022

[22] De Cicco P, Catani MV, Gasperi V, Sibilano M, Quaglietta M, Savini I (2019). Nutrition and breast cancer: a literature review on prevention, treatment and recurrence. Nutrients.

[23] Marín Caro MM, Laviano A, Pichard C (2007). Impact of nutrition on quality of life during cancer. Curr Opin Clin Nutr Metab Care.

[24] Vitolins MZ, Griffin L, Tomlinson WV (2013). Randomized trial to assess the impact of venlafaxine and soy protein on hot flashes and quality of life in men with prostate cancer. J Clin Oncol.

[25] Walsh D, Donnelly S, Rybicki L (2000). The symptoms of advanced cancer: relationship to age, gender, and performance status in 1,000 patients. Support Care Cancer.

[26] Masella R, Malorni W (2017) Gender-related differences in dietary habits. Clin Manag Issues 11(2)

[27] Lin L-Y, Hsu C-Y, Lee H-A (2019). Gender difference in the association of dietary patterns and metabolic parameters with obesity in young and middle-aged adults with dyslipidemia and abnormal fasting plasma glucose in Taiwan. Nutr J.

[28] Kanter R, Caballero B (2012). Global gender disparities in obesity: a review. Adv Nutr.

[29] Alkazemi D (2019). Gender differences in weight status, dietary habits, and health attitudes among college students in Kuwait: a cross-sectional study. Nutr Health.

[30] Schag CAC, Ganz PA, Heinrich RL (1991). Cancer rehabilitation evaluation system–short form (CARES-SF). A cancer specific rehabilitation and quality of life instrument. Cancer.

[31] Te Velde A, Sprangers M, Aaronson N (1996). Feasibility, psychometric performance, and stability across modes of administration of the CARES-SF. Ann Oncol.

[32] Schouten B, De Jonckheere D, Aerts M (2019). An explorative study on systematic assessment of QOL and care needs with the CARES-SF in the early follow-up of patients with digestive cancer. Support Care Cancer.

[33] Ware JE, Kosinski M, Dewey JE, Gandek B (2001). How to score and interpret single-item health status measures: a manual for users of the SF-8 health survey. Lincoln, RI: QualityMetric Incorporated.

[34] Lefante JJ, Harmon GN, Ashby KM, Barnard D, Webber LS (2005). Use of the SF-8 to assess health-related quality of life for a chronically ill, low-income population participating in the Central Louisiana Medication Access Program (CMAP). Qual Life Res.

[35] Gajda R, Jeżewska-Zychowicz M (2020). Elderly perception of distance to the grocery store as a reason for feeling food insecurity—Can food policy limit this?. Nutrients.

[36] Bandi P, Cahn Z, Sauer AG (2021). Trends in E-cigarette use by age group and combustible cigarette smoking histories, US adults, 2014–2018. Am J Prev Med.

[37] Simmons LA, Modesitt SC, Brody AC, Leggin AB (2006). Food insecurity among cancer patients in Kentucky: a pilot study. J Oncol Pract.

[38] Gany F, Melnic I, Wu M, et al (2022) Food to overcome outcomes disparities: a randomized controlled trial of food insecurity interventions to improve cancer outcomes. J Clin Oncol. 10.1200/JCO.21.02400 10.1200/JCO.21.02400 PMC9622577 35709430

[39] Ekwueme DU, Yabroff KR, Guy GP (2014). Medical costs and productivity losses of cancer survivors—United States, 2008–2011. Morb Mortal Wkly Rep.

[40] Dowling EC, Chawla N, Forsythe LP (2013). Lost productivity and burden of illness in cancer survivors with and without other chronic conditions. Cancer.

[41] Baker AH, Wardle J (2003). Sex differences in fruit and vegetable intake in older adults. Appetite.

[42] Arganini C, Saba A, Comitato R, Virgili F, Turrini A (2012). Gender differences in food choice and dietary intake in modern western societies. Public Health-social Behav Health.

[43] Bere E, Brug J, Klepp K-I (2008). Why do boys eat less fruit and vegetables than girls?. Public Health Nutr.

[44] Menon KC (2014). Optimizing nutrition support in cancer care. Asian Pac J Cancer Prev.

